# Review of the Case Reports on Metformin, Sulfonylurea, and Thiazolidinedione Therapies in Type 2 Diabetes Mellitus Patients

**DOI:** 10.3390/medsci11030050

**Published:** 2023-08-15

**Authors:** Elis Susilawati, Jutti Levita, Yasmiwar Susilawati, Sri Adi Sumiwi

**Affiliations:** 1 Doctoral Program in Pharmacy, Faculty of Pharmacy, Padjadjaran University, Sumedang 45363, West Java, Indonesia; elis.susilawati@bku.ac.id; 2 Faculty of Pharmacy, Bhakti Kencana University, Bandung 40614, West Java, Indonesia; 3 Department of Pharmacology and Clinical Pharmacy, Faculty of Pharmacy, Padjadjaran University, Sumedang 45363, West Java, Indonesia; sri.adi@unpad.ac.id; 4 Department of Biology Pharmacy, Faculty of Pharmacy, Padjadjaran University, Sumedang 45363, West Java, Indonesia; yasmiwar@unpad.ac.id

**Keywords:** diabetes mellitus, glucose homeostasis, insulin resistance, pancreatic β-cells, sulfonylureas, thiazolidinediones

## Abstract

Type 2 diabetes mellitus (T2DM) is the world’s most common metabolic disease. The development of T2DM is mainly caused by a combination of two factors: the failure of insulin secretion by the pancreatic β-cells and the inability of insulin-sensitive tissues to respond to insulin (insulin resistance); therefore, the disease is indicated by a chronic increase in blood glucose. T2DM patients can be treated with mono- or combined therapy using oral antidiabetic drugs and insulin-replaced agents; however, the medication often leads to various discomforts, such as abdominal pain, diarrhea or constipation, nausea and vomiting, and hypersensitivity reactions. A biguanide drug, metformin, has been used as a first-line drug to reduce blood sugar levels. Sulfonylureas work by blocking the ATP-sensitive potassium channel, directly inducing the release of insulin from pancreatic β-cells and thus decreasing blood glucose concentrations. However, the risk of the failure of sulfonylurea as a monotherapy agent is greater than that of metformin or rosiglitazone (a thiazolidinedione drug). Sulfonylureas are used as the first-line drug of choice for DM patients who cannot tolerate metformin therapy. Other antidiabetic drugs, thiazolidinediones, work by activating the peroxisome proliferator-activated receptor gamma (PPARγ), decreasing the IR level, and increasing the response of β-cells towards the glucose level. However, thiazolidines may increase the risk of cardiovascular disease, weight gain, water retention, and edema. This review article aims to discuss case reports on the use of metformin, sulfonylureas, and thiazolidinediones in DM patients. The literature search was conducted on the PubMed database using the keywords ‘metformin OR sulfonylureas OR thiazolidinediones AND case reports’, filtered to ‘free full text’, ‘case reports’, and ‘10 years publication date’. In some patients, metformin may affect sleep quality and, in rare cases, leads to the occurrence of lactate acidosis; thus, patients taking this drug should be monitored for their kidney status, plasma pH, and plasma metformin level. Sulfonylureas and TZDs may cause a higher risk of hypoglycemia and weight gain or edema due to fluid retention. TZDs may be associated with risks of cardiovascular events in patients with concomitant T2DM and chronic obstructive pulmonary disease. Therefore, patients taking these drugs should be closely monitored for adverse effects.

## 1. Introduction

The WHO defines diabetes mellitus (DM) as a chronic metabolic disease that is characterized by an increase in blood glucose and causes defects to the heart, blood vessels, kidneys, and nerves [1,2]. The International Diabetes Federation (IDF) recently reported that of approximately 415 million DM patients, 91% were categorized as T2DM [3]. Moreover, Indonesian Basic Health Research 2018 reported that the prevalence of T2DM in Indonesia had increased remarkably [4]. Patients with T2DM tend to have 15% higher mortality risks compared to those without DM [5,6].

More than 90% of DM cases are type 2 (T2DM), which is mainly caused by a combination of two factors: the failure of insulin secretion by the pancreatic β-cells and the inability of insulin-sensitive tissues to respond to insulin (insulin resistance) [3,5]. Insulin resistance (IR) is a condition whereby insulin-targeted tissues, e.g., adipose, liver, and muscle, encounter metabolic disorders, thus leading to metabolic syndrome diseases, non-alcoholic fatty liver disease (NAFLD), atherosclerosis, and T2DM [7,8,9].

T2DM is a metabolic and endocrine disorder initiated by complex interactions between genetic factors, unhealthy lifestyle, and pancreatic β-cell dysfunction, along with other endocrine abnormalities [10,11,12]. T2DM is always associated with IR, a condition whereby pancreatic β-cells fail to compensate for insulin [13,14,15], leading to a continuous increase in blood glucose; thus, the demand for more insulin release is needed [3,16]. The distorted function of insulin induces the elevation of lipogenesis but fails to inhibit gluconeogenesis [17]. Gluconeogenesis is the specific characteristic of IR and T2DM: the elevation of hepatic glucose production whereby glycogen synthesis in the liver keeps maintaining the gluconeogenic activity without decreasing the high level of blood glucose [14,17]. The accumulation of fat in the liver eventually escalates the blood glucose levels through the reduction in insulin sensitivity and the occurrence of IR [18]. IR stimulates the increase in fatty acids in the blood, reduces the transportation of glucose to the muscle cells, and increases lipolysis, thus leading to the rise in hepatic glucose production [19,20].

The most effective management of T2DM needs an adjustment in both lifestyles, including a healthy diet and routine workouts, and oral antidiabetic drugs [21]. This review article aims to comprehensively discuss the case reports on metformin, sulfonylurea, and thiazolidinedione therapies in T2DM patients. Briefly, a literature search was conducted on the PubMed database (https://pubmed.ncbi.nlm.nih.gov/ accessed from 10 January to 30 May 2023) using the keywords ‘metformin OR sulfonylureas OR thiazolidinediones AND case reports’, filtered to ‘free full text’, ‘case reports’, and ’10 years publication date’. The initial search resulted in 496 articles, which were further screened by the title and the abstract for their relevance to the topic of interest. Review articles, systematic reviews, and clinical trials were excluded. The case report articles included in this review totaled four for sulfonylureas and two for thiazolidinediones; for metformin, only the cases that involved nightmares were included in this review. An additional search was conducted on Google using the same keywords.

## 2. Pharmacology of Sulfonylureas

Sulfonylureas are classified as both first- (e.g., tolbutamide and chlorpropamide) and second-generation antidiabetic drugs (e.g., glyburide, gliclazide, glipizide, and glimepiride). The second generation of anti-DM drugs has shown higher potency with a lower dose [22]. Both generations are able to significantly diminish glycosylated hemoglobin (HbA1c) levels. HbA1c represents the blood glucose status over the previous 90 days [23,24].

Sulfonylureas work by blocking the ATP-sensitive potassium channel, directly inducing the release of insulin from pancreatic β-cells, thus decreasing blood glucose concentrations [7,25]. However, the failure levels of sulfonylureas as monotherapy drugs are greater compared to those of metformin (biguanide) or rosiglitazone (thiazolidinedione). Sulfonylureas are the first-line drugs of choice for patients who cannot tolerate metformin [22].

More than 90% of sulfonylureas in the blood are bound to the plasma proteins, which eventually cause drug–drug interactions with salicylates, sulfonamides, and warfarin [22]. The main side effects of sulfonylureas are a higher risk of hypoglycemia and weight gain [26]. The increase in BW is associated with the anabolic effect of higher insulin levels and the decrease in glycosuria [14,15]. Several cases of the use of sulfonylureas in DM patients are summarized in Table 1.

Sulfonylureas are the first-line drugs of choice for patients who cannot tolerate metformin. These drugs may cause mild to severe adverse events in T1DM or T2DM patients [27,28,29,30]. However, it should be noted that although oral hypoglycemic drugs are not recommended during pregnancy, sulfonylureas can be used in gestational diabetes mellitus with a normal outcome of the pregnancy because hyperglycemia often occurs during organogenesis and is linked with an elevated risk of congenital malformations, preeclampsia, premature delivery, intrauterine infections, and perinatal mortality [27].

## 3. Pharmacology of Thiazolidinediones

Pioglitazone and rosiglitazone, which are both thiazolidinediones (TZDs), work by activating the peroxisome proliferator-activated receptor gamma (PPARγ) [31], decreasing the IR level, and increasing the response of β-cells towards the glucose level [22,32,33]. However, thiazolidines may increase the risk of cardiovascular disease [26], weight gain, water retention, and edema [14]. Pioglitazone (15 mg/day) effectively reduced aminotransferase and bilirubin levels in a nonalcoholic steatohepatitis patient within three months of therapy [31]. Rosiglitazone has been withdrawn due to its effect on the bones and because it may cause an ischemic heart attack [33]. The most reported side effect of pioglitazone is the occurrence of pleural effusion in diabetic patients [34]. Several cases of the use of thiazolidinediones in DM patients are presented in Table 2.

It was estimated that 1.5–2.6% of cases experienced a worsening of DME after TZD (pioglitazone and/or rosiglitazone) therapy. Fluid retention was observed in 5–15% of patients treated with glitazones, and drug discontinuation resulted in the rapid resolution of both peripheral and macular edema [37]. TZDs may directly alter water reabsorption in the kidneys by affecting tubular transport, sodium retention, and vascular hyperpermeability or indirectly by affecting renal hemodynamics [38]. It was shown that the exposure of primary collecting duct cells to TZDs induced an increase in sodium transport, body weight gain, and plasma volume expansion [39].

Moreover, the prevalence of bladder cancer in T2DM patients treated with pioglitazone was recently studied. All the patients (n = 6440) were of Asian Indian ethnicity. It was concluded that pioglitazone is not correlated with a bladder cancer risk. However, this drug should not be given to patients with a history of hematuria [40].

The meta-analysis of randomized control trials revealed that TZDs had significantly decreased HbA1c, fasting blood glucose, and elevated HDL levels [41]. In a retrospective analysis, TZD administered as an add-on to metformin was reported safe for patients with mild renal impairment and normal renal function [42]. Another study reported that rosiglitazone therapy was related to a high risk of major cardiovascular events. In patients with concomitant T2DM and chronic obstructive pulmonary disease, TZDs may be associated with more risks of cardiovascular events, ventilation use, pneumonia, and lung cancer [43].

All things considered, thiazolidine pioglitazone has advantages in terms of the decreasing of triglycerides, the increasing of HDL, and the decreasing of the LDL concentration, which is thought to be due to the activation of PPAR-α [44].

## 4. Pharmacology of Metformin

Metformin (dimethyl biguanide) has been used to reduce blood sugar levels; however, other guanidines (phenformin and buformin) were withdrawn due to a lactate acidosis risk [22,45].

Metformin or Glucophage is the first-line therapy for T2DM management. This drug has been approved by the FDA (Food and Drug Administration) [8,45,46,47]. Metformin ameliorates glycemic control in patients with T2DM by amending insulin sensitivity in the liver. The main outcome is a decrease in glucose synthesis in the liver and an elevation in glucose excretion in the skeletal muscles [25,47,48]. The oral bioavailability of metformin is 40–60% with T1/2 plasma of 4–9 h. T2DM patients treated with metformin usually experience a reduction in the fasting plasma glucose of 2–4 mmol/L and in HbA1c of 1–2%, independently of their age, body weight, and the duration of therapy [49]. The main side effects of metformin therapy are nausea and other gastrointestinal inconveniences [22]. A correlation was reported between long-term metformin use and vitamin B12 deficiency [50,51,52]. Metformin causes depression, numbness, vision disorders, and mouth ulcers [52]. Although metformin is the oral drug of choice for patients with T2DM, several cases of the use of metformin that involved nightmares have been reported globally and are summarized in Table 3.

A cross-sectional study of more than 200 patients with childhood-onset T1DM reported that 26% of individuals had nightmares and 41% of them had poor sleep quality. The participants with uncontrolled glycemia revealed a higher frequency of nightmares compared to the patients with better glycemic control [56].

It was decided that the nocturnal decrease in cerebral blood glucose levels was the cause of the occurrence of nightmares and abnormal dreams [57]. The occurrence of abnormal dreams and the low quality of sleep among T2DM patients were found to be associated with poor glycemic control in such patients [58]. Studies in humans suggested that the endocannabinoid system was overactivated in patients with obesity and/or DM. The activation of the cannabinoid-1 receptors stimulates weight gain and is associated with metabolic changes [59]. Moreover, in a recent study, the HbA1c level was reported to correlate with the duration of sleep in T2DM patients. It was suggested that patients with T2DM should have an appropriate range of sleep duration [60].

In addition to sleep disorders, metformin therapy may also cause lactic acidosis. A case of a 70-year-old male Caucasian diagnosed with T2DM who had taken a deliberate metformin overdose revealed a profound lactic acidosis with a pH of 6.93 and a lactate level > 20 mmol/L. The patient was admitted to the ICU and was given continuous hemodiafiltration with an average blood flow rate of 150 mL/hour for 48 h and 8.4% sodium bicarbonate to neutralize the acid [61].

A recent study also reported 4241 cases of lactic acidosis in T2DM patients who were metformin users. Metformin-associated lactic acidosis (MaLA), although it rarely occurs, has been of great concern due to its life-threatening prevalence. Key information to have when evaluating a case of suspected MaLA includes the times and doses of metformin medication, the kidney condition, plasma pH, concentrations of metformin in the plasma, and details of concomitant drugs and comorbid risk factors for lactate acidosis [62]. A very rare case of metformin-induced hemolytic anemia was reported in a 17-year-old male patient [63].

There are very few cases of metformin use reported in Indonesia. However, a recent case of metformin in Lampung, Sumatra, is described as follows: a female patient (65 years old) was diagnosed with T2DM 2 years ago and had complaints of fatigue accompanied by weakness during activities. The fatigue was rarely felt, but it later became more frequent. The complaints were not accompanied by blurred vision or numbness. This patient had an HbA1C of 8.7%. It should be noted that for elderly T2DM patients, lifestyle adjustment is critical as a first step [64]. Metformin has been reported to cause a deficiency of vitamin B12 [45,65,66].

The safety of long-term use of metformin in 393 T2DM patients with contraindications has been studied. Of them, 67.68% were suffering from coronary heart disease, and 23.92% were from congestive heart failure. It was concluded that the patients who tolerate the drug well may continue taking it, even when mild kidney impairment evolves [67].

## 5. Conclusions

**Figure 1:**
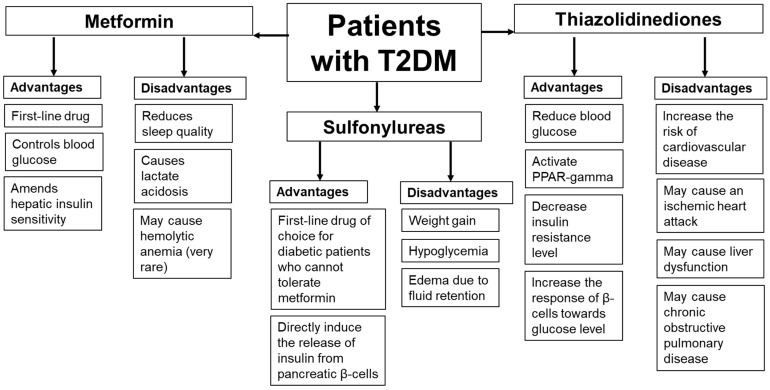
The advantages and disadvantages of metformin, sulfonylurea, and thiazolidinedione therapies based on the case reports.

Metformin is the first-line therapy for patients with T2DM. This drug ameliorates glycemic control by amending insulin sensitivity in the liver. However, data about the metformin side effects are limited and based mainly on case reports. It should be noted that in some patients, metformin may affect sleep quality by inducing abnormal nightmares and, in rare cases, the occurrence of lactate acidosis. Patients taking this drug should be monitored for their kidney status, plasma pH, and plasma metformin level. Another antidiabetic drug, sulfonylurea, is used as the first-line drug of choice for DM patients who cannot tolerate metformin therapy. Other antidiabetic drugs, TZDs, work by activating the PPAR-γ, decreasing the IR level, and increasing the response of β-cells toward the glucose level. However, it should be noted that in some patients, sulfonylureas and TZDs may cause a higher risk of hypoglycemia and weight gain or edema due to fluid retention. TZDs may be associated with risks of cardiovascular events in patients with concomitant T2DM and chronic obstructive pulmonary disease. Therefore, patients taking these drugs should be closely monitored for adverse effects. Based on the case reports, the advantages and disadvantages of these drugs are summarized in Figure 1.

## Figures and Tables

**Table 1:** Cases of the use of sulfonylureas in DM patients.

Presentation of the Cases in T1DM Patients	Treatment	Outcome	Ref.
PNDM ^1^ female patient, a carrier of the R201H mutation. Treated with MDI ^2^ of insulin since infancy (6 weeks old; HbA1c level was maintained at 7.2%). Replaced insulin therapy with sulfonylureas (GITS ^3^) at 34 years old (HbA1c decreased to 5.9%). The patient was pregnant at 37 years old while she was on 30 mg of GITS (HbA1c 5.8%) with a congenital abnormality of the uterus.	GITS was discontinued and replaced with MDI of insulin and one injection of NPH ^4^ insulin.	Recurrent episodes of hypoglycemia and hyperglycemia occurred at week 9 (HbA1c 5.9%). The patient was hospitalized, given sulfonylurea (glibenclamide 40 mg/day) until the delivery, and treated with small doses of prandial insulin when moderate hyperglycemia arose after meals. HbA1c level in week 29 was 5.8%. Proteinuria in week 34. Premature caesarean delivery took place in week 35; the baby’s weight was 3010 g, with phenotypic features of diabetic fetopathy. Normalization of the baby’s glucose level occurred on day 3. It was concluded that although oral hypoglycemic drugs are not recommended during pregnancy, sulfonylureas can be used in gestational diabetes mellitus with a normal outcome of the pregnancy.	[27]
A 28-year-old Irish female with PNDM ^1^, treated with subcutaneous MDI of insulin from 4 weeks of age. At the age of 16 years, she was diagnosed with a KCNJ11 gene mutation (R201H genotype).	At the age of 16, she replaced insulin therapy with sulfonylureas (glibenclamide), 1 mg/kg/day, experienced nausea after taking glibenclamide, and was treated with Motilium. Insulin therapy was never fully discontinued due to a persistent increase in glucose levels. She started on insulin Detemir, 4 units twice/day. She was given glibenclamide, 15 mg twice/day.	Glibenclamide maintained the patient’s blood sugar levels to normal, with infrequent episodes of hypoglycemia (HbA1c of 44 mmol/mol for 2 years).	[28]
**Presentation of the** **Cases in T2DM Patients**			
A 64-year-old female T2DM patient with recurrent severe hypoglycemic episodes for the previous few months. She was on 80 mg of immediate-release gliclazide two times/day for years and was not adherent due to the hypoglycemic episodes. The patient suffered from chronic kidney disease and severe aortic stenosis. On arrival, the blood glucose level was 2.5 mmol/L. She experienced a 5 kg weight loss in spite of a good appetite, no change in bowel habits, abdominal pain, recent febrile episodes, headaches, and chest pain.	The patient was administered 100 mL of 10% dextrose IV for initial treatment and was hospitalized. During hospitalization, she experienced multiple episodes of spontaneous hypoglycemia: blood glucose 2 mmol/L, serum cortisol 285 nmol/L, and increased levels of proinsulin, insulin, C-peptide, and decreased level of β-hydroxybutyrate. She was started on a continuous 5% dextrose IV infusion at 84 mL/hour to prevent further hypoglycemic episodes and her gliclazide was omitted.	On day 21 after gliclazide discontinuation, the levels of proinsulin, insulin, and C-peptide decreased, while the level of β-hydroxybutyrate elevated. Considering the chronic kidney disease and severe sulphonylurea-induced hypoglycemia, the patient was treated with a daily dose of insulatard at 5 units/day. The blood glucose was satisfactory without further episodes of hypoglycemia and hyperglycemia, and she was eventually deemed fit for discharge with regular outpatient follow-up appointments.	[29]
A 56-year-old male T2DM patient with new onset hypoglycemia. He was taking oral metformin (2500 mg/day in divided doses), gliclazide modified release (90 mg/day), and an MDI ^2^ insulin (insulin glargine, 18 units subcutaneously at night, and insulin lispro, 6 units subcutaneously with meals). The patient was adherent to all medications and had never developed hypoglycemia. He had a bone marrow transplantation for myelofibrosis 8 months prior and long-term treatment with prednisone (5 mg orally/day), resulting in an immunocompromised state. One week earlier, he was diagnosed with fungal pneumonia and was given voriconazole (200 mg orally twice/day). Due to voriconazole intolerance, his antifungal was switched to fluconazole (480 mg orally/day). After starting fluconazole, he stopped his insulin due to hypoglycemic episodes and felt weak, confused, and lethargic.	The patient was treated with a 50 mL bolus of 50% dextrose followed by a 100 mL/hour infusion of 10% dextrose. For the sulfonylurea intoxication, the patient was given octreotide, 75 μg subcutaneously, and the IV dextrose was discontinued as his blood sugar normalized. One further dose of octreotide was given 6 h later.	The hypoglycemic episodes of the patient stopped with no recurrence during hospitalization. The patient’s hypoglycemia in the setting of azole antifungal co-prescription raised a concern regarding gliclazide toxicity due to an adverse drug interaction.	[30]

^1^ PNDM: permanent neonatal diabetes mellitus (or type 1 DM); ^2^ MDI: multiple daily injections; ^3^ GITS: glipizide gastrointestinal therapeutic system; ^4^ NPH: neutral protamine Hagedorn, intermediate-acting insulin used in the treatment of DM.

**Table 2:** Cases of the use of thiazolidinediones in DM patients.

Presentation of the Patient	Treatment	Outcome	Ref.
A 66-year-old female patient with AN ^1^ with liver dysfunction not due to alcohol or hepatitis. CT ^2^ and USG ^3^ indicated normal liver. BMI ^4^: 11.1 kg/m^2^ Body temperature: 37.4 °C BP: 120/64 mmHg HR: 97 beats/minute O_2_ saturation: 98% Cardiovascular, pulmonary, abdominal, and neurological examinations were normal. The skin was dry with decreased turgor. Serum AST ^5^ and ALT ^6^ levels were 3665 IU/L and 1495 IU/L, respectively. Serological tests for HBsAg and anti-HCVAb were negative. Prothrombin time was 52%. Serum bilirubin, creatinine, and urea nitrogen were elevated. Thyroid hormone levels were normal.	TPN ^7^ with the addition of phosphorus was initiated at 1000 kcal/day. One hundred milliliters of SNMC ^8^ was administered daily to improve the AST and ALT levels but was discontinued on day 8 due to secondary aldosteronism, resulting in increases in AST and ALT levels. SNMC treatment was resumed on day 13 and the serum AST and ALT levels decreased. Total bilirubin levels increased on day 20. To rule out liver infection prior to the administration of prednisolone (PSL), a percutaneous needle biopsy of the liver was conducted on day 21 and resulted in a diagnosis of secondary NASH ^9^ with AN. Thus, 40 mg PSL and 900 mg UDCA ^10^ were administered daily, and 15 mg pioglitazone was administered daily, starting on day 31.	Liver function was completely normalized on day 105. The liver function remained normal after SNMC and PSL treatment were tapered off, and the UDCA dosage was reduced to 300 mg per day. The patient became able to walk following physical rehabilitation and was discharged on day 168.	[32]
A 30-year-old female patient with T2DM (HbA1c of 12%) and moderate non-proliferative diabetic retinopathy. Mild vitreous hemorrhage was observed in both eyes, but the retinopathy was stable with proliferative tissues surrounding the optic disc. DME ^11^ did not develop during or immediately after the pan-retinal photocoagulation in both eyes. Two weeks after taking pioglitazone, the patient’s face was edematous; she had gained weight and severe DME in both eyes and a serous retinal detachment in the right eye.	Pioglitazone was discontinued and 25 mg of spironolactone to reduce fluid retention and peripheral edema was administered.	Two months later, the patient’s BW was reduced by 8 kg, and her DME was significantly improved in both eyes. Her final visual acuity returned to 0.9 OD and 0.7 OS.	[35]
A 16-year-old female T2DM patient with polyphagia, polydipsia, and polyuria during the previous 7 months. She was treated with 1500 mg/day metformin and 60 IU/day insulin (insulin aspart injection of 10 IU at breakfast, 10 IU at lunch, and 10 IU at dinner, insulin degludec of 30 IU at bedtime). Blood glucose: 10–14 mmol/L (fasting) and 12–18 mmol/L (postprandial). HbA1c 8.4%.	The insulin doses were reduced to 29 IU/day and combined with oral hypoglycemic drug (30 mg/day pioglitazone and 1500 mg/day metformin), medication for protecting the liver (75 mg/day bicyclol), and medication for reducing proteinuria (5 mg/day benazepril).	The fasting and postprandial blood glucose levels were decreased 9–10 mmol/L and 10–13 mmol/L, respectively. She was discharged to home with instructions for outpatient follow-up. After 2 months of follow-up, she complained of asthenia and night sweats without hypoglycemia.	[36]

^1.^ AN: anorexia nervosa; ^2^ CT: computed tomography; ^3^ USG: ultrasonography; ^4^ BMI: body mass index; ^5^ AST: aspartate aminotransferase; ^6^ ALT: alanine aminotransferase; ^7^ TPN: total parenteral nutrition; ^8^ SNMC: Stronger Neo-Minophagen C (Minophagen Pharmaceutical Co., Ltd., Tokyo, Japan); ^9^ NASH: nonalcoholic steatohepatitis; ^10^ UDCA: ursodeoxycholic acid; ^11^ DME: diabetic macular edema.

**Table 3:** Cases of the use of metformin in T2DM Patients.

Presentation of the Patient	Treatment	Outcome	Ref.
A newly diagnosed 40-year-old Asian male T2DM patient reported recurrent nightmares within a week of starting his metformin medication. This patient was normotensive and a nonsmoker, with a happy marriage and a settled job. The HbA1c of the patient was 7.3%, and the random blood glucose was 295 mg/dL. He was given an initial regular-release dose of metformin, 500 mg 2 × 1 tablet/day, to be taken in conjunction with a healthy diet and lifestyle.	Metformin was discontinued and replaced with a GLP-1 agonist.	The patient reported no more nightmares on the discontinuation of metformin.	[53]
A 56-year-old male T2DM patient with no known history of recurrent nightmares or sleep disorders reported having nightmares and abnormal dreams directly after taking his 750 mg extended-release metformin. This patient had an HbA1c of 7.6% and was overweight, but his liver enzymes, serum creatinine, and urea were within normal ranges.	Metformin was temporarily discontinued. On the night when metformin was retaken, the abnormal dreams returned. It was suggested that the patient should take metformin in the morning instead of at night.	Morning metformin administration decreases nightmares.	[54]
A 30-year-old male T2DM patient, prescribed with a combination of sitagliptin and metformin (50 mg/500 mg) twice daily was reported to have had abnormal nightmares beginning after a week of metformin therapy. The patient did not smoke or drink alcohol. HbA1c: 7.2% Blood glucose: 110 (fasting) and 190 mg/dL (postprandial)	Metformin was stopped and the therapy was substituted with a combination of 100 mg of sitagliptin and 0.5 mg of glimepiride daily.	On the 10th day after metformin discontinuation, the patient reported no nightmares or abnormal dreams. After 2 months, metformin was reintroduced and the patient again experienced nightmares and abnormal dreams.	[55]

## Data Availability

Not applicable.
